# Supplementary figures and images for: The structure of species discrimination signals across a primate radiation (part 2 of 2)
Source: eLife. 2020 Jan 13;9:e47428. doi: 10.7554/eLife.47428 (PMC6957270; doi:10.7554/eLife.47428)

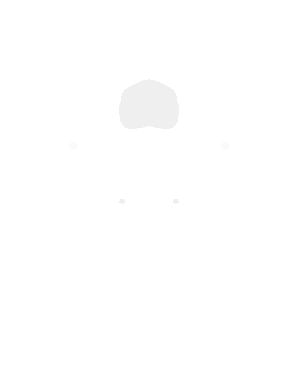

Supplement: Supplementary file 1. [file elife-47428-supp1.zip › Supplementary_File_1/mona_monkey_subset/species_mean_occluder/right_hemiface/C_sclateri.tiff]

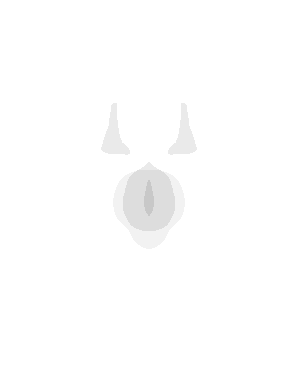

Supplement: Supplementary file 1. [file elife-47428-supp1.zip › Supplementary_File_1/mona_monkey_subset/species_mean_occluder/right_hemiface/Ch_tantalus.tiff]

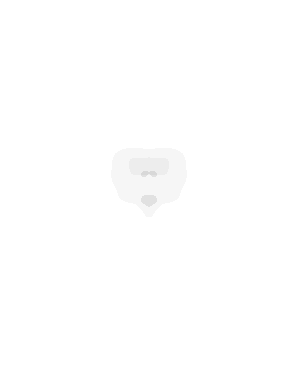

Supplement: Supplementary file 1. [file elife-47428-supp1.zip › Supplementary_File_1/mona_monkey_subset/species_mean_occluder/right_hemiface/E_patas.tiff]

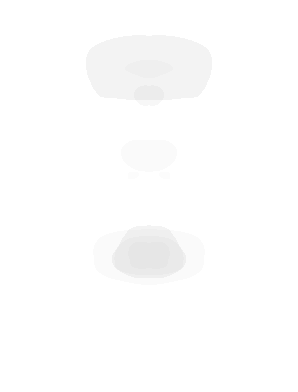

Supplement: Supplementary file 1. [file elife-47428-supp1.zip › Supplementary_File_1/putty_nosed_monkey_subset/grey_occluder/left_hemiface/A_nigroviridis.tiff]

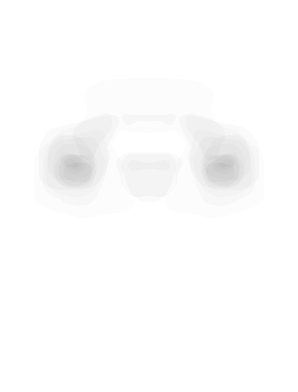

Supplement: Supplementary file 1. [file elife-47428-supp1.zip › Supplementary_File_1/putty_nosed_monkey_subset/grey_occluder/left_hemiface/C_ascanius.tiff]

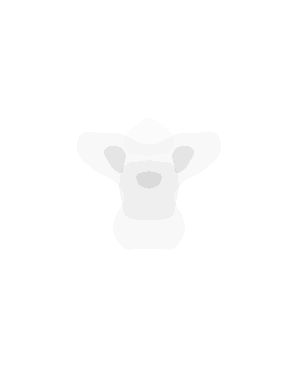

Supplement: Supplementary file 1. [file elife-47428-supp1.zip › Supplementary_File_1/putty_nosed_monkey_subset/grey_occluder/left_hemiface/C_diana.tiff]

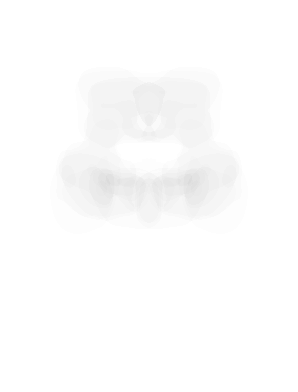

Supplement: Supplementary file 1. [file elife-47428-supp1.zip › Supplementary_File_1/putty_nosed_monkey_subset/grey_occluder/left_hemiface/C_mona.tiff]

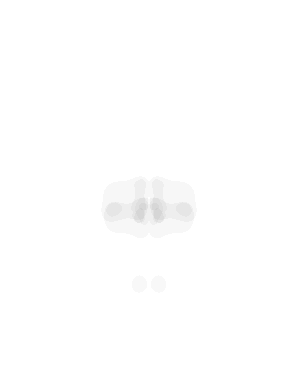

Supplement: Supplementary file 1. [file elife-47428-supp1.zip › Supplementary_File_1/putty_nosed_monkey_subset/grey_occluder/left_hemiface/C_neglectus.tiff]

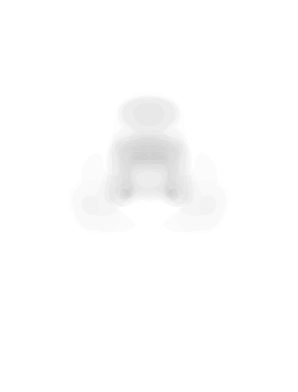

Supplement: Supplementary file 1. [file elife-47428-supp1.zip › Supplementary_File_1/putty_nosed_monkey_subset/grey_occluder/left_hemiface/C_nictitans.tiff]

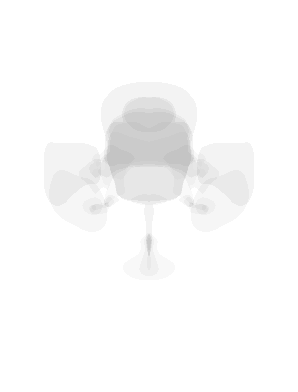

Supplement: Supplementary file 1. [file elife-47428-supp1.zip › Supplementary_File_1/putty_nosed_monkey_subset/grey_occluder/left_hemiface/C_petaurista.tiff]

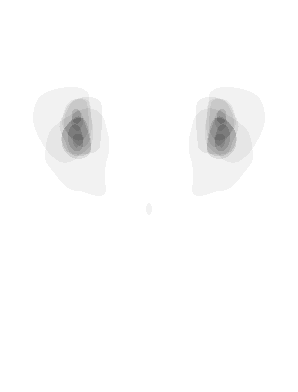

Supplement: Supplementary file 1. [file elife-47428-supp1.zip › Supplementary_File_1/putty_nosed_monkey_subset/grey_occluder/left_hemiface/Ch_tantalus.tiff]

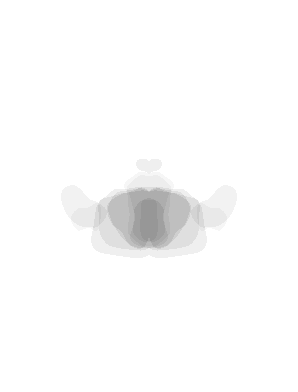

Supplement: Supplementary file 1. [file elife-47428-supp1.zip › Supplementary_File_1/putty_nosed_monkey_subset/grey_occluder/left_hemiface/E_patas.tiff]

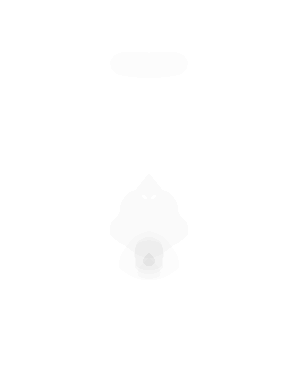

Supplement: Supplementary file 1. [file elife-47428-supp1.zip › Supplementary_File_1/putty_nosed_monkey_subset/grey_occluder/right_hemiface/A_nigroviridis.tiff]

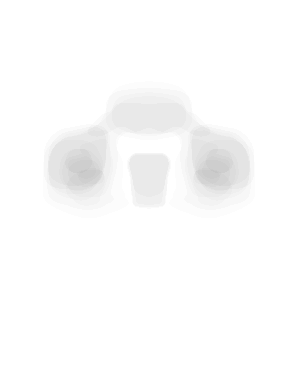

Supplement: Supplementary file 1. [file elife-47428-supp1.zip › Supplementary_File_1/putty_nosed_monkey_subset/grey_occluder/right_hemiface/C_ascanius.tiff]

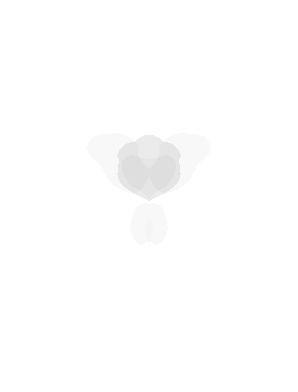

Supplement: Supplementary file 1. [file elife-47428-supp1.zip › Supplementary_File_1/putty_nosed_monkey_subset/grey_occluder/right_hemiface/C_diana.tiff]

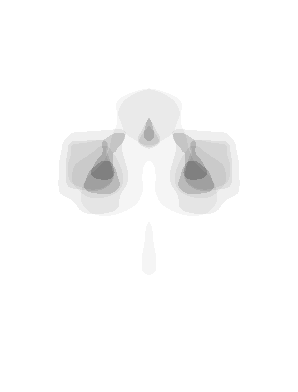

Supplement: Supplementary file 1. [file elife-47428-supp1.zip › Supplementary_File_1/putty_nosed_monkey_subset/grey_occluder/right_hemiface/C_erythrotis.tiff]

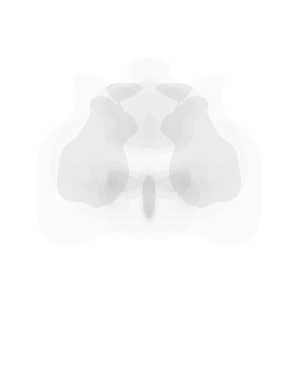

Supplement: Supplementary file 1. [file elife-47428-supp1.zip › Supplementary_File_1/putty_nosed_monkey_subset/grey_occluder/right_hemiface/C_mona.tiff]

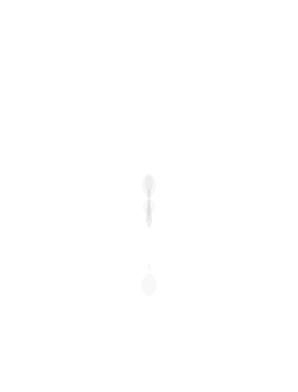

Supplement: Supplementary file 1. [file elife-47428-supp1.zip › Supplementary_File_1/putty_nosed_monkey_subset/grey_occluder/right_hemiface/C_neglectus.tiff]

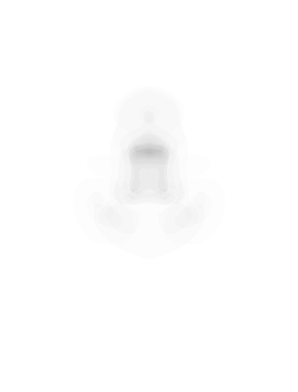

Supplement: Supplementary file 1. [file elife-47428-supp1.zip › Supplementary_File_1/putty_nosed_monkey_subset/grey_occluder/right_hemiface/C_nictitans.tiff]

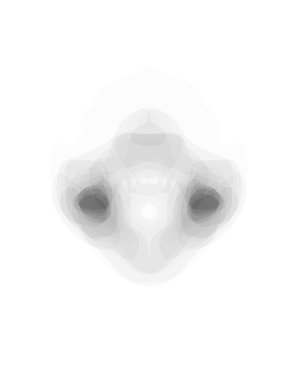

Supplement: Supplementary file 1. [file elife-47428-supp1.zip › Supplementary_File_1/putty_nosed_monkey_subset/grey_occluder/right_hemiface/C_petaurista.tiff]

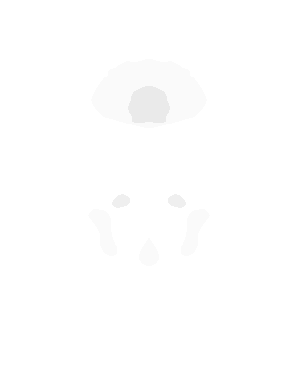

Supplement: Supplementary file 1. [file elife-47428-supp1.zip › Supplementary_File_1/putty_nosed_monkey_subset/grey_occluder/right_hemiface/C_sclateri.tiff]

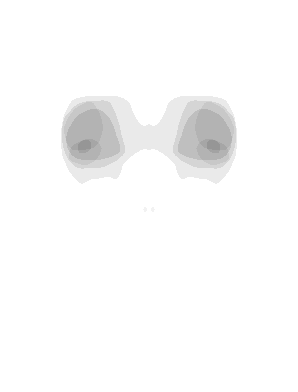

Supplement: Supplementary file 1. [file elife-47428-supp1.zip › Supplementary_File_1/putty_nosed_monkey_subset/grey_occluder/right_hemiface/Ch_tantalus.tiff]

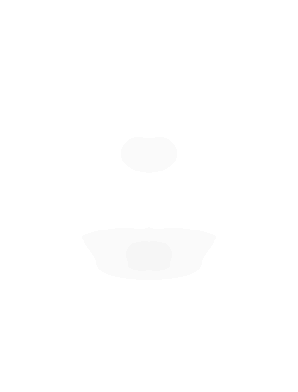

Supplement: Supplementary file 1. [file elife-47428-supp1.zip › Supplementary_File_1/putty_nosed_monkey_subset/species_mean_occluder/left_hemiface/A_nigroviridis.tiff]

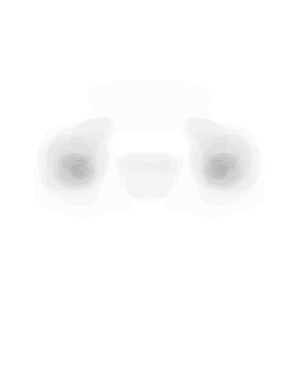

Supplement: Supplementary file 1. [file elife-47428-supp1.zip › Supplementary_File_1/putty_nosed_monkey_subset/species_mean_occluder/left_hemiface/C_ascanius.tiff]

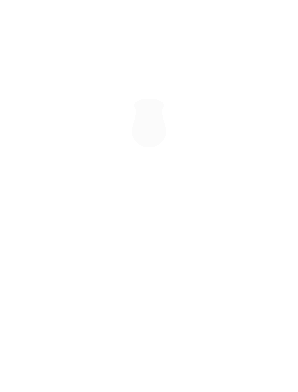

Supplement: Supplementary file 1. [file elife-47428-supp1.zip › Supplementary_File_1/putty_nosed_monkey_subset/species_mean_occluder/left_hemiface/C_diana.tiff]

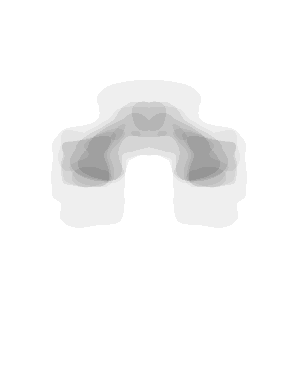

Supplement: Supplementary file 1. [file elife-47428-supp1.zip › Supplementary_File_1/putty_nosed_monkey_subset/species_mean_occluder/left_hemiface/C_erythrotis.tiff]

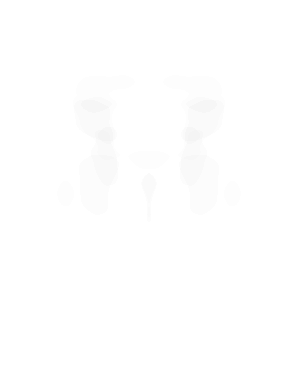

Supplement: Supplementary file 1. [file elife-47428-supp1.zip › Supplementary_File_1/putty_nosed_monkey_subset/species_mean_occluder/left_hemiface/C_mona.tiff]

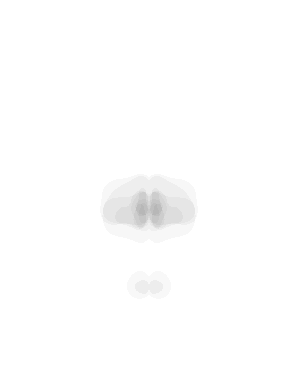

Supplement: Supplementary file 1. [file elife-47428-supp1.zip › Supplementary_File_1/putty_nosed_monkey_subset/species_mean_occluder/left_hemiface/C_neglectus.tiff]

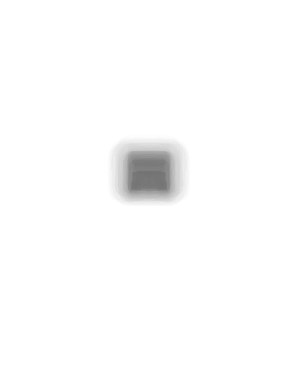

Supplement: Supplementary file 1. [file elife-47428-supp1.zip › Supplementary_File_1/putty_nosed_monkey_subset/species_mean_occluder/left_hemiface/C_nictitans.tiff]

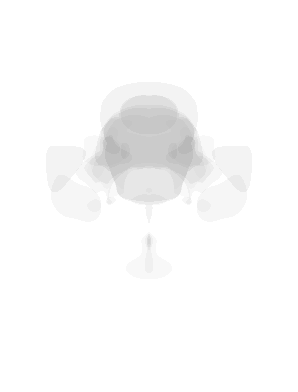

Supplement: Supplementary file 1. [file elife-47428-supp1.zip › Supplementary_File_1/putty_nosed_monkey_subset/species_mean_occluder/left_hemiface/C_petaurista.tiff]

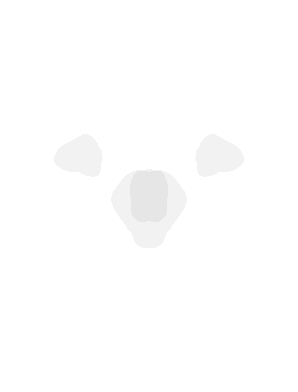

Supplement: Supplementary file 1. [file elife-47428-supp1.zip › Supplementary_File_1/putty_nosed_monkey_subset/species_mean_occluder/left_hemiface/Ch_tantalus.tiff]

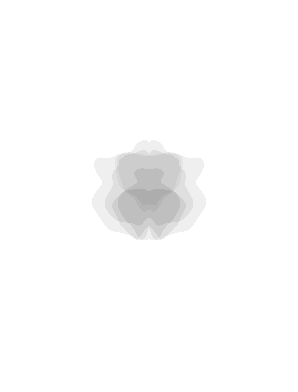

Supplement: Supplementary file 1. [file elife-47428-supp1.zip › Supplementary_File_1/putty_nosed_monkey_subset/species_mean_occluder/left_hemiface/E_patas.tiff]

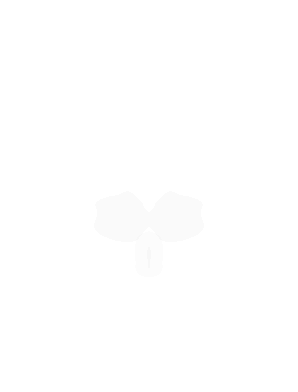

Supplement: Supplementary file 1. [file elife-47428-supp1.zip › Supplementary_File_1/putty_nosed_monkey_subset/species_mean_occluder/right_hemiface/A_nigroviridis.tiff]

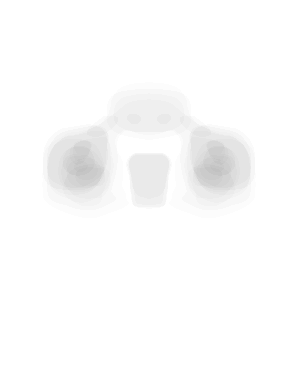

Supplement: Supplementary file 1. [file elife-47428-supp1.zip › Supplementary_File_1/putty_nosed_monkey_subset/species_mean_occluder/right_hemiface/C_ascanius.tiff]

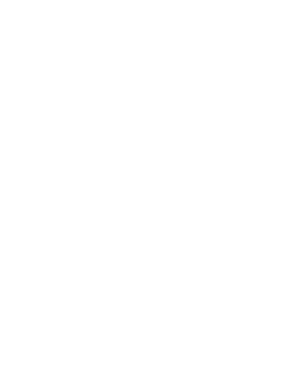

Supplement: Supplementary file 1. [file elife-47428-supp1.zip › Supplementary_File_1/putty_nosed_monkey_subset/species_mean_occluder/right_hemiface/C_diana.tiff]

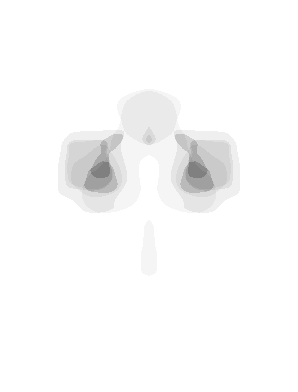

Supplement: Supplementary file 1. [file elife-47428-supp1.zip › Supplementary_File_1/putty_nosed_monkey_subset/species_mean_occluder/right_hemiface/C_erythrotis.tiff]

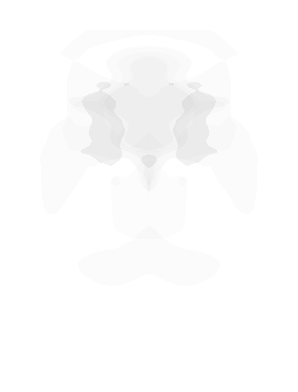

Supplement: Supplementary file 1. [file elife-47428-supp1.zip › Supplementary_File_1/putty_nosed_monkey_subset/species_mean_occluder/right_hemiface/C_mona.tiff]

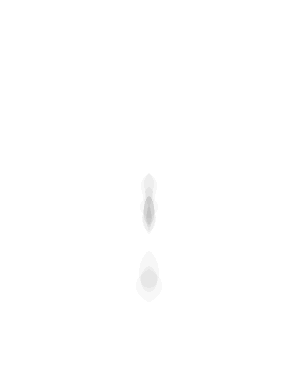

Supplement: Supplementary file 1. [file elife-47428-supp1.zip › Supplementary_File_1/putty_nosed_monkey_subset/species_mean_occluder/right_hemiface/C_neglectus.tiff]

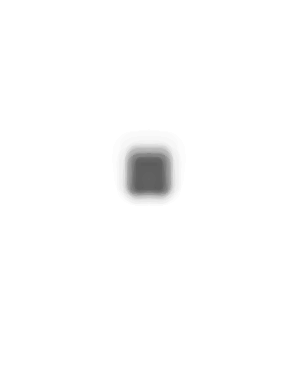

Supplement: Supplementary file 1. [file elife-47428-supp1.zip › Supplementary_File_1/putty_nosed_monkey_subset/species_mean_occluder/right_hemiface/C_nictitans.tiff]

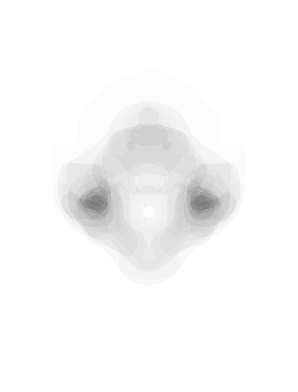

Supplement: Supplementary file 1. [file elife-47428-supp1.zip › Supplementary_File_1/putty_nosed_monkey_subset/species_mean_occluder/right_hemiface/C_petaurista.tiff]

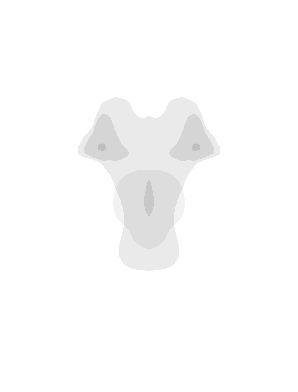

Supplement: Supplementary file 1. [file elife-47428-supp1.zip › Supplementary_File_1/putty_nosed_monkey_subset/species_mean_occluder/right_hemiface/Ch_tantalus.tiff]

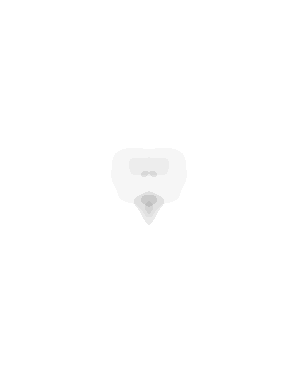

Supplement: Supplementary file 1. [file elife-47428-supp1.zip › Supplementary_File_1/putty_nosed_monkey_subset/species_mean_occluder/right_hemiface/E_patas.tiff]
