# Supplementary material for: The structure of species discrimination signals across a primate radiation
Source: eLife. 2020 Jan 13;9:e47428. doi: 10.7554/eLife.47428 (PMC6957270; doi:10.7554/eLife.47428)
Supplement: Supplementary file 2. [file elife-47428-supp2.docx]

Supplementary Materials: Looking time results

# Table 1

Results from model comparisons for putty nosed monkeys. The final model is the best fit to the data and was used to make factor-level comparisons.

| **Model** | **Fixed effects** | **Comparison** | **Likelihood ratio test** |  |
| --- | --- | --- | --- | --- |
| M1 | None | n/a | n/a |  |
| M2 | Species (con- v. heterospecific) | M1 | Chisq = 3.051, df = 1, p = 0.081 | **∙** |
| M3^*^ | Trait (shared v. not shared) | M1 | Chisq = 10.473, df = 1, p = 0.001 | ** |
| M4 | Subject sex (male v. female) | M1 | Chisq = 0, df = 1, p = 1 |  |
| M5 | Subject age (numerical) | M1 | Chisq = 0, df = 1, p = 1 |  |
| M6 | Subject origin (captive v. wild) | M1 | Chisq = 0, df = 1, p = 1 |  |
| M7^*^ | Presentation side (right v. left) | M1 | Chisq = 17.697, df = 1, p < 0.001 | *** |
| M8 | Eye contact (yes v. no) | M1 | Chisq = 0.185, df = 1, p = 0.667 |  |
| M9 | Familiarity (ordinal: not present < present but not visible < visible) | M1 | Chisq = 3.878, df = 2, p = 0.144 |  |
| M10 | Stimulus sex (male v. female) | M1 | Chisq = 0, df = 1, p = 1 |  |
| M11 | Trial order (categorical: first, second, third) | M1 | Chisq = 0, df = 2, p = 1 |  |
| M12 | Apparatus pattern (4 categories) | M1 | Chisq = 0, df = 3, p = 1 |  |
| M13 | ICC profile (5 categories) | M1 | Chisq = 0, df = 1, p = 1 |  |
|  |  |  |  |  |
| M14^*^ | Species + trait + presentation side | M1 | Chisq = 31.933, df = 3, p < 0.001 | *** |
| M15 | Trait + presentation side^1^ | M14 | Chisq = 3.055, df = 1, p = 0.081 | **∙** |
| M16 | Species + presentation side^1^ | M14^*^ | Chisq = 11.511, df = 1, p < 0.001 | *** |
| M17 | Species + trait^1^ | M14^*^ | Chisq = 18.065, df = 1, p < 0.001 | *** |
|  |  |  |  |  |
| Final^*^ | Trait + presentation side | M1 | Chisq = 28.878, df = 1, p < 0.00 | *** |

^*^Denotes which of the two models was a better fit to the data when the difference was statistically significant.

^1^Model comparisons test the significance of the factor that was removed from the expanded model (e.g. comparing M15 to M14 tests the significance of species).

# Table 2

Results from model comparisons for mona monkeys. The final model is the best fit to the data and was used to make factor-level comparisons.

| **Model** | **Fixed effects** | **Comparison** | **Likelihood ratio test** |  |
| --- | --- | --- | --- | --- |
| M1 | None | n/a | n/a |  |
| M2^*^ | Species (con- v. heterospecific) | M1 | Chisq = 179.640, df = 1, p < 0.001 | *** |
| M3^*^ | Trait (shared v. not shared) | M1 | Chisq = 31.621, df = 1, p < 0.001 | *** |
| M4 | Subject sex (male v. female) | M1 | Chisq = 0, df = 1, p = 1 |  |
| M5 | Subject age (numerical) | M1 | Chisq = 0, df = 1, p = 1 |  |
| M6 | Subject origin (captive v. wild) | M1 | Chisq = 0, df = 1, p = 1 |  |
| M7 | Presentation side (right v. left) | M1 | Chisq = 0.985, df = 1, p = 0.321 |  |
| M8 | Eye contact (yes v. no) | M1 | Chisq = 0.761, df = 1, p = 0.383 |  |
| M9 | Stimulus sex (male v. female) | M1 | Chisq = 0, df = 1, p = 1 |  |
| M10 | Trial order (categorical: first, second, third) | M1 | Chisq = 0, df = 2, p = 1 |  |
| M11 | Apparatus pattern (4 categories) | M1 | Chisq = 0, df = 3, p = 1 |  |
| M12 | ICC profile (4 categories) | M1 | Chisq = 0, df = 3, p = 1 |  |
|  |  |  |  |  |
| M13^*^ | Species + trait | M1 | Chisq = 209.100, df = 2, p < 0.001 | *** |
| M14 | Trait^1^ | M13^*^ | Chisq =177.480, df = 1, p < 0.001 | *** |
| M15 | Species^1^ | M13^*^ | Chisq = 29.462, df = 1, p < 0.001 | *** |
| M16^*^ | Species*trait + species + trait | M13 | Chisq = 8.242, df = 1, p = 0.004 | ** |
|  |  |  |  |  |
| Final^*^ | Species*trait + species + trait | M1 | Chisq = 217.340, df = 3, p < 0.001 | *** |

^*^Denotes which of the two models was a better fit to the data when the difference was statistically significant.

^1^Model comparisons test the significance of the factor that was removed from the expanded model (e.g. comparing M14 to M13 tests the significance of species).
